# Colloidal transport and flocculation are the cause of the hyperenrichment of gold in nature

**DOI:** 10.1073/pnas.2100689118

**Published:** 2021-05-11

**Authors:** Duncan F. McLeish, Anthony E. Williams-Jones, Olga V. Vasyukova, James R. Clark, Warwick S. Board

**Affiliations:** ^a^Department of Earth and Planetary Sciences, McGill University, Montréal, QC, Canada H3A 0E8;; ^b^Pretium Resources Inc., Vancouver, BC, Canada V7X 1L4

**Keywords:** gold, colloidal transport, epithermal systems, hydrothermal fluid, Brucejack deposit

## Abstract

Aqueous complexation has long been considered the only viable means of transporting gold to depositional sites in hydrothermal ore-forming systems. A major weakness of this hypothesis is that it cannot readily explain the formation of ultrahigh-grade gold veins. This is a consequence of the relatively low gold concentrations typical of ore fluids (tens of parts per billion [ppb]) and the fact that these “bonanza” veins can contain weight-percent levels of gold in some epithermal and orogenic deposits. Here, we present direct evidence for a hypothesis that could explain these veins, namely, the transport of the gold as colloidal particles and their flocculation in nanoscale calcite veinlets. These gold-bearing nanoveinlets bear a remarkable resemblance to centimeter-scale ore veins in many hydrothermal gold deposits and give unique insight into the scale invariability of colloidal flocculation in forming hyperenriched gold deposits. Using this evidence, we propose a model for the development of bonanza gold veins in high-grade deposits. We argue that gold transport in these systems is largely mechanical and is the result of exceptionally high degrees of supersaturation that preclude precipitation of gold crystals and instead lead to the formation of colloidal particles, which flocculate and form much larger masses. These flocculated masses aggregate locally, where they are seismically pumped into fractures to locally form veins composed largely of gold. This model explains how bonanza veins may form from fluids containing ppb concentrations of gold and does not require prior encapsulation of colloidal gold particles in silica gel, as proposed by previous studies.

## Significance

Hydrothermal veins supply much of the Earth’s gold. Their propensity to contain “bonanza” occurrences of gold that have concentrations millions of times greater than the concentration of gold in Earth’s crust makes them important targets for exploration and resource development. The mechanisms by which such hyperenrichment occurs are enigmatic. The accepted wisdom is that this enrichment reflects the saturation and precipitation of gold from hydrothermal fluids. Laboratory experiments and measurements of active hydrothermal systems, however, have shown that the solubility of this noble metal is exceptionally low. Here, we demonstrate that this issue is resolved by the physical transport of gold in the solid state as nanoparticles and their flocculated aggregates, thereby explaining the paradox of bonanza gold ore formation.

**Fig. 1.**
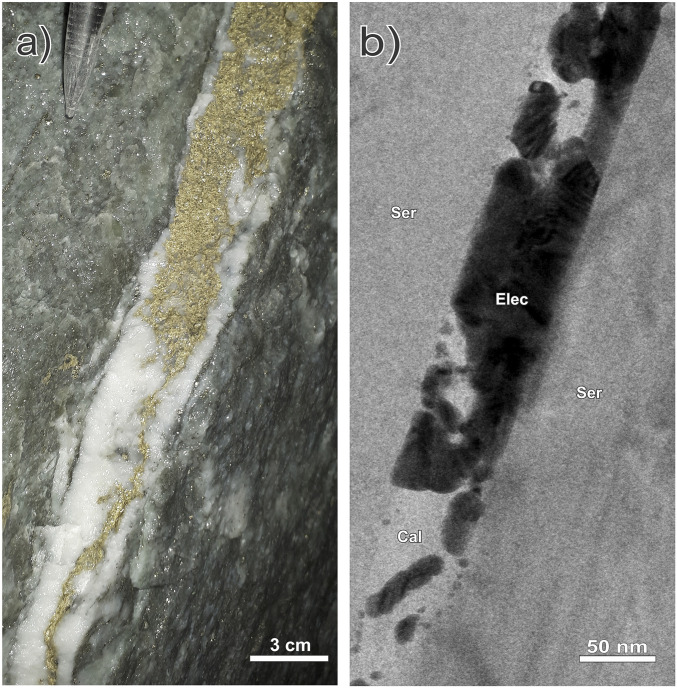
Ultrahigh-grade (bonanza) electrum mineralization in (*A*) a calcite-quartz vein hosted by sericitized crystal tuff in a development face in the underground mine workings and (*B*) in a calcite nanoveinlet (bright-field TEM image). The two images emphasize the strong similarity between massive (clotted) electrum at the macro and nano scales. Spherical nanoparticles of electrum are evident in the bottom left corner of the nanoveinlet. The tip of a mechanical pencil appears in the top left corner of *A* for scale.

Measurements of gold concentrations in the fluids responsible for epithermal mineralization and in geothermal fluids, which are considered to be analogs of epithermal ore fluids, are on the order of 10 to 30 parts per billion (ppb) (1). Moreover, similar concentrations have been calculated for fluids of appropriate composition from the results of experiments designed to determine the speciation of gold in aqueous fluids (2). Although such concentrations may be sufficient to form veins containing tens of grams per tonne of gold, they are far too low to explain spatially discrete occurrences of ultrahigh-grade concentrations which, in some veins, exceed 50 wt % of gold per tonne on the decimeter scale; these bonanza intervals are commonly accompanied by intervals in the same vein containing <5 ppm gold (Fig. 1). Formation of such bonanza veins by direct precipitation of native gold or electrum from the ore fluids would require that individual fractures remain open for unreasonably long periods of time [>>50,000 y (1), which is a timeframe that exceeds the total lifespan of many porphyry–epithermal deposits (3) and greatly exceeds the estimated ∼1,400 y required to seal a 1-m-wide vein grading 20 g/t Au in an active geothermal system (4)] or that the fluid flux be extraordinarily high. Indeed, a recent study by Pearce et al. (5) suggests that the fluid fluxes are typically quite low. They estimated a fluid:rock ratio of ∼12:1 and concluded from gold solubility considerations that the maximum amount of gold per tonne of rock that could be deposited for this ratio is 20 g.

The existence of gold colloids has been known since the mid-nineteenth century (6), and the idea that gold deposits might form from fluids transporting the gold as a colloid (i.e., solid nanoparticles with charged surfaces dispersed in an electrolyte solution) was proposed more than 80 y ago (7–9). Demonstration, however, that gold can be dissolved in chloride- and bisulfide-bearing aqueous fluids in concentrations of tens and even hundreds of parts per million (2, 10, 11) has led to widely accepted genetic models in which the gold is transported to the site of deposition exclusively as dissolved species and precipitates in crystalline form (12). Nonetheless, there have been some advocates of colloidal transport, notably Petrovskaya and Kvasnifsa et al. (13, 14), who presented textural evidence at millimeter to micrometer scales in support of a model, in which gold nanoparticles deposit and aggregate to form dendrites. Noting that gold dendrites are commonly hosted by quartz, Saunders (15, 16), Saunders and Schoenly (17), and Herrington and Wilkinson (18) proposed that the transport of colloidal gold particles was facilitated by the occurrence of flocculated colloidal silica or silica gel. They did not, however, provide direct evidence for the presence of gold colloids.

More recently, Hannington et al. (19), Gartman et al. (20), Hannington and Garbe-Schönberg (21), and Prokofiev et al. (22) provided indirect evidence for the existence of gold colloids in hydrothermal fluids. Hannington et al. detected gold particles ranging from 2 μm to less than 50 nm in diameter in geothermal fluids and seafloor black smokers and showed that the mass of particulate and dissolved gold in bulk samples of the fluid greatly exceeds that predicted from solubility calculations. Prokofiev et al. detected high concentrations of gold in fluid inclusions, up to 6,000 ppm Au, which they interpreted to represent gold nanoparticles. The only studies that have provided images of colloidal gold particles are those of Harrichhausen (23), Saunders and Burke (24), Burke et al. (25), and Petrella et al. (26). These four studies proposed a model of gold concentration similar to that of Saunders (15) in which visible (centimeter-scale) gold dendrites in hydrothermal veins are the products of recrystallization of gold colloid–bearing amorphous silica. None of these studies have provided direct evidence for colloidal gold flocculation in nanoveinlets and neither have they shown that this phenomenon can potentially occur at the scale of a bonanza-type deposit.

## The Brucejack Gold Deposit

In order to test for the existence of gold nanoparticles in a fossil hydrothermal system and gain insight into the process by which they could form bonanza gold veins, we have undertaken a transmission electron microscopy (TEM) study of mineralization from the Brucejack epithermal gold deposit in northwestern British Columbia, Canada. This deposit, which was emplaced near the margin of a long-lived island arc at ∼184 Ma (27), is host to abundant, spatially restricted occurrences of bonanza-grade mineralization [up to 41,582 g/t Au in a 0.5 m diamond drill core interval (28)] in veins with an average thickness of 10 cm (ranging up to a maximum thickness of 2 m). Local concentrations of >1,000 g/t Au occur in at least three generations of electrum-bearing quartz-carbonate veins over a vertical extent exceeding 1,500 m which, from fluid inclusion studies, are interpreted to have developed as a result of boiling and/or fluid mixing (28). The high-grade gold mineralization occurs typically as 1 to 30 cm diameter/length dense clots, comprising microveinlets of electrum [Au_62_Ag_38_, on average (29)] and calcite in narrow (1 to 50 cm wide) vein swarms and wider (meter- to decameter-scale) quartz-carbonate stockworks and vein breccias. The distribution of electrum clots within the veins is highly irregular; some veins contain many clots in close proximity to each other, such that the veins locally contain over 50 wt % electrum (Fig. 1), whereas others contain a single electrum clot. Moreover, within vein swarms commonly only a single vein contains electrum clots, and the others are visibly barren. The wallrock to both the electrum-bearing and barren veins has been affected strongly by extensive, preelectrum phyllic alteration, which hosts lower grade (<5 g/t Au), invisible gold in colloform, oscillatory arsenic-rich growth zones in pyrite (29). These rocks, however, do not contain electrum except in rare cases where localized postmineral deformation sheared electrum along fault planes beyond vein confines.

## Methods and Results

**Fig. 2.**
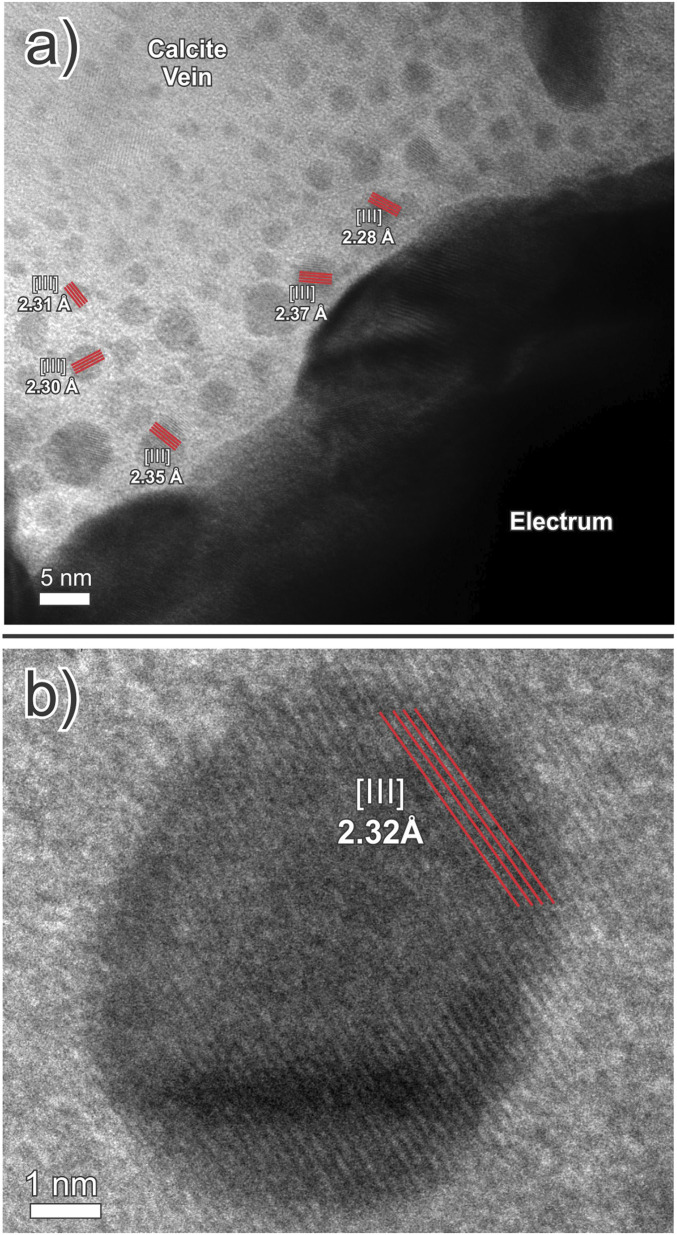
Bright-field TEM images of the contact between a large electrum grain and the calcite matrix in a calcite-quartz vein. (*A*) Abundant spherical electrum nanoparticles in calcite and (*B*) an electrum nanoparticle with a lattice fringe spacing of 2.32 Å.

**Fig. 3.**
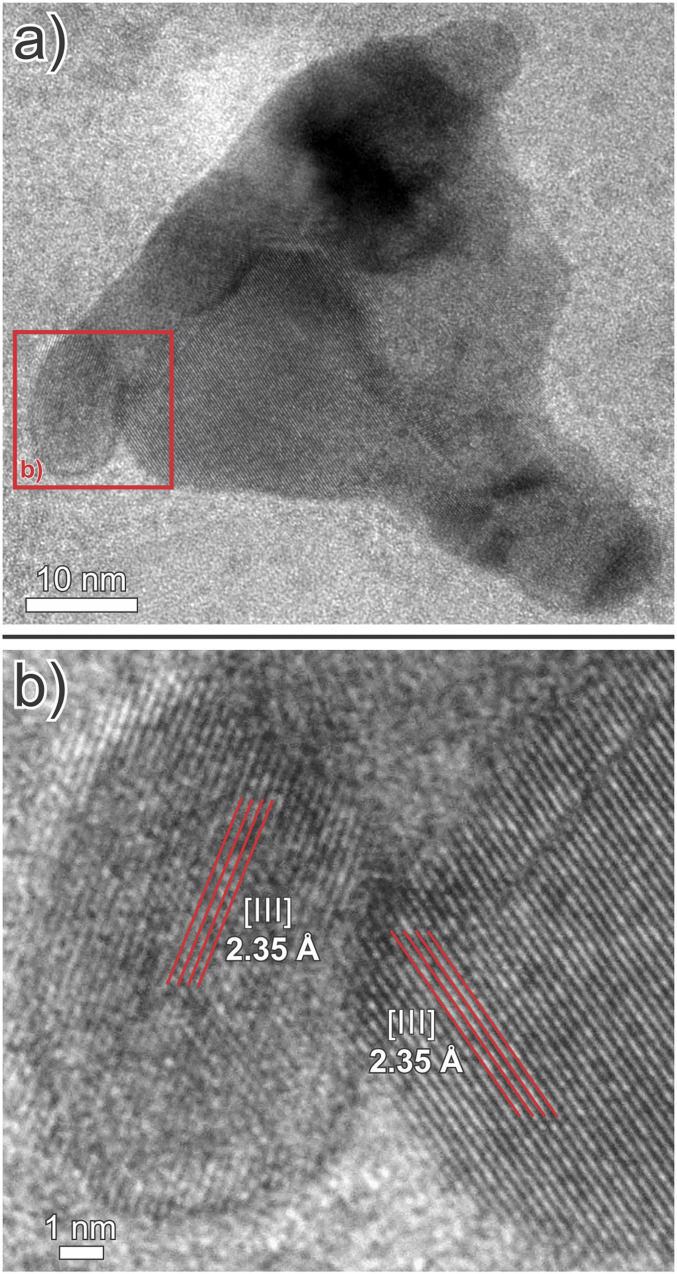
(*A*) A bright-field TEM image of a large electrum nanoparticle. (*B*) An enlargement of the nanoparticle in *A* showing that it is composed of numerous, ovoid to spherical nanoparticles with variable lattice plane orientations.

**Fig. 4.**
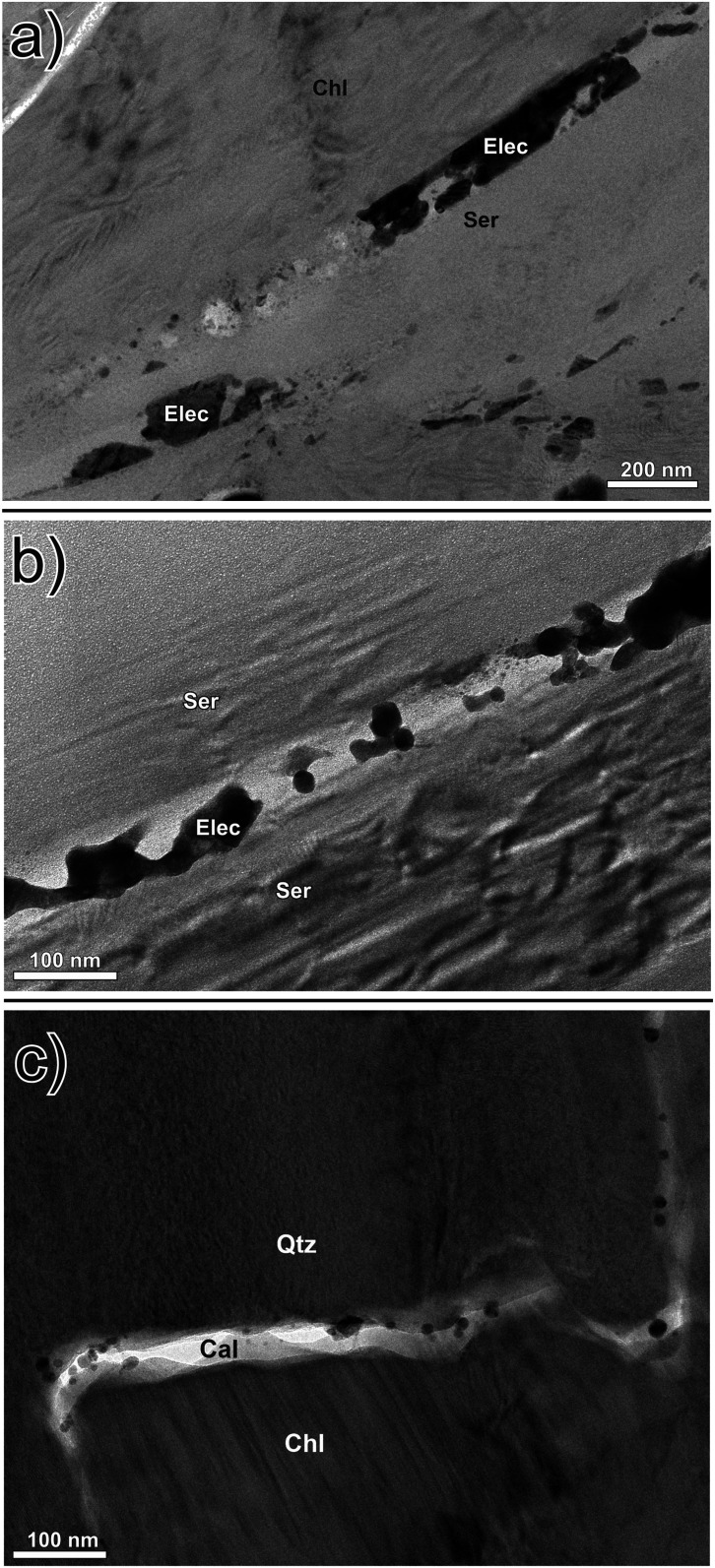
(*A*) A bright-field TEM image of electrum nanoparticles (occurring as both flocculated masses and individual nanoparticles) within calcite nanoveinlets that exploited cleavage planes in chlorite and sericite (this is a wider field of view version of Fig. 1*B*). (*B*) A calcite nanoveinlet containing flocculated and individual spherical electrum nanoparticles that occupies a cleavage plane in sericite. (*C*) Electrum nanoparticles in a nanoveinlet of calcite developed along a grain boundary between chlorite and quartz; the nanoveinlet also cuts quartz.

We prepared five ultrathin (50 to 100 nm) TEM lamellae from dendritic clots of electrum in two samples of calcite-quartz vein stockworks from different locations in the Brucejack mine to investigate the nature of the mineralization at the nanoscale. These stockworks are typical of those hosting bonanza-grade electrum occurrences throughout the mine. The TEM lamellae were thinned using the focused ion beam milling technique and were centered on areas of electrum in contact with vein-hosted calcite and quartz (see *SI Appendix* for analytical details). Our resulting bright-field TEM images (e.g., Figs. 1*B* and 2–4) document the existence of numerous dark spheres, ∼5 to 15 nm in diameter, which are disseminated in the calcite matrix of 0.5 to 1 cm wide veins or concentrated in calcite-bearing nanoveinlets (<100 nm wide). In the example illustrated in Fig. 2*A*, they occur adjacent to a large grain of electrum and exhibit crystal lattice fringes with a spacing of 2.2 to 2.4 Å (Fig. 2*B*), which is the same as the spacing in the adjacent electrum grain, as well as that of experimentally produced gold and electrum nanoparticles [2.4 and 2.2 Å, respectively (30)]. Nanoparticles similar in size to those shown in Fig. 2*A* are present in all the lamellae, and in all cases where lattice fringes could be observed the lattice spacing was found to be in the range 2.2 to 2.4 Å. There are also larger particles of electrum in all the lamellae, ∼30 to 150 nm in diameter (Fig. 3*A*), which consist of multiple 5 to 10 nm wide domains displaying variable lattice plane orientations (Fig. 3*B*). We interpret these larger particles to be aggregates of nanoparticles, with each nanoparticle having a different lattice plane orientation to that of the adjacent nanoparticles. In all the lamellae, the matrix to the disseminated electrum nanoparticles has a lattice fringe spacing of 3.0 to 3.1 Å, which is the same as that for calcite [∼3.0 Å (31)] and distinctly different from that of quartz [∼3.4 Å (32)]. Although quartz was observed in some lamellae, it was not found to contain nanoparticles of electrum.

One of the lamellae, from the contact of a mineralized vein with wallrock, contains electrum nanoparticle-bearing nanoveinlets of calcite (50 to 100 nm wide). These nanoveinlets exploited the cleavage planes of chlorite and sericite (Fig. 4 *A* and *B*) and are developed along the boundaries between chlorite and quartz crystals or crosscut the quartz (Fig. 4*C*). The electrum occurs either as isolated particles or aggregates of particles that occupy the full width of the nanoveinlets for distances up to 600 nm (Fig. 4*A*).

## Discussion

Although the formation of 1 to 5 nm negatively charged gold nanoparticles (colloidal gold) and their flocculation has been demonstrated in laboratory experiments (8, 33–36), this study provides direct evidence of the existence of flocculated colloidal-scale gold nanoparticles and documents their transport in nanoscale hydrothermal veinlets in a natural system. Specifically, we have shown that isolated, spherical 1 to 5 nm electrum nanoparticles, identified by their lattice fringe spacing, electron diffraction maxima, and energy-dispersive X-ray spectroscopic (EDS) spectra (see *SI Appendix* for indexed selected area electron diffraction images and EDS spectra), are common in the Brucejack deposit and that they form aggregates composed of multiple spherical to ovoid subparticles with unique lattice plane orientations. The irregular shapes exhibited by the larger aggregates are strikingly similar to the dendritic habit displayed by coarse-grained clots of electrum in veins at Brucejack. This mimicry of electrum textures from the nano to the macro scale is consistent with the fractal nature of colloidal aggregates observed in experimental studies, as well as in computer simulations of the flocculation of gold colloids (e.g., ref. 37).

Numerous TEM studies of experimentally grown synthetic crystals (e.g., anatase) have demonstrated that minerals may commonly go through a nanoparticle phase during the incipient stage of crystallization that accompanies precipitation from solution (e.g., refs. 38 and 39). The nanoparticle phase is generally short lived, with crystals continuing to grow beyond this stage by Ostwald ripening. In nonequilibrium systems (e.g., boiling hydrothermal systems, in which there is extreme supersaturation due to very steep gradients in physicochemical parameters such as temperature), however, Ostwald ripening is not a viable mechanism for crystal growth because nucleation rates are too high and growth rates are too low (40). In such cases, atoms have insufficient time to organize themselves into crystals and, instead, accumulate in spherical masses to minimize surface area for a given volume. As nonequilibrium environments can persist throughout the evolution of a porphyry–epithermal hydrothermal system (e.g., at boiling/fluid mixing sites), conditions conducive to the formation of metallic nanoparticles may prevail locally for the same duration. The exact location(s) of nanoparticle formation in a hydrothermal system, and the distances over which nanoparticles may be transported in suspension, are currently unknown but, for gold nanoparticles, solubility considerations dictate that the nucleation sites are likely to be in the deeper parts of hydrothermal systems (i.e., in the upper levels of the porphyry environment) where physicochemical conditions permit greater gold solubility yet vigorous episodic boiling is still possible.

The flocculation of metallic nanoparticles from a hydrothermal fluid can be achieved through boiling, cooling, and/or fluid mixing. Boiling acts in two separate yet reinforcing ways to promote flocculation: 1) the physical effects of boiling increase the collision efficiency of nanoparticles; and 2) boiling-related pH increases lead to the precipitation of minerals containing cationic flocculants (e.g., Al^3+^ and Fe^3+^), which “bridge” negatively charged nanoparticles (15, 41). Cooling slows the reduction of Au^+^ to Au^0^ and decreases the number of nucleation centers for gold nanoparticles, which could lead to the development of larger nanoparticles that flocculate more easily (8, 42). The mixing of seawater (see below), which is moderately alkaline (pH 7.5 to 8.5), with an acidic to near-neutral hydrothermal fluid, favors the precipitation of cationic flocculants due to the increase in pH but also adds cationic flocculants (e.g., Na^+^) and accelerates cooling, which in turn promotes flocculation (15). Several lines of evidence suggest that all these processes (boiling, cooling, and fluid mixing) were active during the formation of ultrahigh-grade gold veins at Brucejack. Tombe et al. (28), on the basis of a detailed study of fluid inclusions in quartz from two generations of electrum-bearing quartz-calcite veins, concluded that the fluid boiled during vein formation. In addition, the widespread occurrence of hydrothermal breccias and the abundance of auriferous, oscillatory-zoned arsenian pyrite with strongly negative δ^34^S signatures [e.g., −40.25‰ (43)] is strong evidence of boiling, which oxidizes the fluid by releasing hydrogen, thereby fractionating the heavier isotope into sulfate and the light isotope into reduced sulfur. Similar evidence of boiling has been cited for other epithermal gold deposits [e.g., Porgera, Papua New Guinea (44, 45)]. During the final stage of pyrite crystallization (outer zones of crystals), which was synchronous with electrum flocculation, δ^34^S values increased sharply from strongly negative to between +20.0 and +24.0‰ (43). Significantly, electrum is locally in textural equilibrium with this latest phase of pyrite crystallization. As the Brucejack deposit was emplaced near the margin of a long-lived island arc in the early Jurassic, and these values, including those of the corresponding fluid, are within the range of those for early Jurassic seawater [∼+15 to 24‰ (46)], we propose that after vigorous, episodic boiling, the hydrothermal ore fluids mixed with seawater. Furthermore, given its close temporal association with electrum deposition, we suggest that this fluid mixing was the principal cause of flocculation and ultimately the reason for the bonanza-style gold mineralization that characterizes the deposit.

We submit, here, a model for the colloidal precipitation, transport, and deposition of electrum, which accounts for the extreme concentrations of gold in quartz-carbonate veins at Brucejack. In this model, electrum colloids form in response to boiling of a hydrothermal fluid and are then mechanically transported to sites where they mix with seawater and flocculate. More specifically, the model involves vigorous boiling in response to strong decompression in the porphyry environment and ascent of the fluids to the shallow crustal level of the Brucejack deposit [≤1,000 m depth (28)] where boiling continued and there was mixing with seawater. The boiling-induced rapid cooling and increases in pH and *f*O_2_ caused extreme supersaturation of gold and the formation of colloidal particles. Whether this boiling also induced flocculation is unknown, although, in principle, the increased particle collision efficiency due to boiling could have promoted the initial flocculation of the electrum nanoparticles (15). We propose, however, that most of the flocculation occurred in response to the mixing of seawater, which was introduced at a late stage of hydrothermal activity and coincided with electrum mineralization. This flocculation at numerous, spatially discrete sites spread throughout the deposit led to the localized clogging of fluid pathways (veins) with massive but sporadically distributed clots of electrum, causing the localized hyperenrichment of gold in the deposit. Seismic pumping resulting from episodic, earthquake-related fault fracturing (47) evidenced by crack-and-fill textures in the multistage mineralized veins and hydrothermal breccias at Brucejack (28), likely helped to circulate flocculated electrum particles through the deposit. By repeatedly clogging the fractures with electrum (as illustrated in Fig. 1*B*) then unclogging them and introducing new particles, this seismic pumping progressively converted the fractures into hyperenriched gold veins and was the final and critical driver of bonanza gold ore formation at Brucejack.

Our model offers a simple solution to the long-standing problem of how hydrothermal fluids with low gold concentrations can produce ultrahigh-grade or bonanza-type gold deposits, a paradox that has not been explained by genetic models involving the in situ precipitation of crystalline gold, and also does not require the prior encapsulation of colloidal gold particles in silica gel as proposed by some previous studies (e.g., refs. 15–18, 23–26). In addition, boiling-induced colloid formation, followed by colloidal transport to sites of fluid mixing-induced flocculation, may help explain the distribution of high-grade gold mineralization over vertical extents (e.g., >1,500 m) that greatly exceed those of the boiling horizons in most fossil hydrothermal systems and their modern geothermal analogs (18, 48). This is because colloid flocculation can occur far from the initial site of colloid formation, whereas the standard boiling model for epithermal deposits requires that phase separation and gold deposition are spatially coincident. We further speculate that the flocculated gold moved through micro- and nanofractures in the main fluid pathways (which became infilled veins), intermittently clogging these fractures as illustrated in Fig. 1*B*, and eventually forming the clots of electrum that, on a much larger, visible scale, appear to have clogged the veins that characterize the bonanza mineralization at Brucejack (Fig. 1*A*). This scenario explains why the massive electrum is texturally late in all the mineralized veins and why the clotted nature of the gold mineralization, though ubiquitous, is texturally variable throughout the deposit.

In conclusion, the recent discoveries in modern hydrothermal systems of gold nanoparticles that may have formed from colloidal suspensions (21), coupled with our evidence for the colloidal transport of gold in a fossil system, suggest that mechanical enrichment of gold may be a much more widespread phenomenon than has been recognized to date. Indeed, this mineralizing process may have occurred in several other types of systems, notably orogenic gold deposits (49), and it may be the key to understanding the formation of ultrahigh-grade or bonanza gold deposits in whatever geological environments they may occur.

## Supplementary Material

Supplementary File

## Data Availability

All study data are included in the article and/or *SI Appendix*.
