# Supplementary material for: Colloidal transport and flocculation are the cause of the hyperenrichment of gold in nature
Source: Proc Natl Acad Sci U S A. 2021 May 11;118(20):e2100689118. doi: 10.1073/pnas.2100689118 (PMC8157991; doi:10.1073/pnas.2100689118)
Supplement: Supplementary File [file pnas.2100689118.sapp.pdf]

Data Supplement #1– Analytical procedures

The TEM lamellae investigated in this study were prepared with a FEI Helios Nanolab 660 DualBeam focused ion beam scanning electron microscope (FIBSEM; Thermo Scientific, Hillsboro, OR USA) at the Facility for Electron Microscopy Research at McGill University using trench and in situ lift-out techniques. The FIBSEM is equipped with a gallium ion source operating in the accelerating-voltage range 0.5 - 30 keV, a gas injection system (GIS) and an EasyLift Nanomanipulator System.

Prior to inserting a thin section into the Helios, an ex-situ layer of Pt was deposited over the surface using the Leica EM ACE600 sputter coater (Leica Microsystems, Wetzlar, Germany). Areas of the thin section containing Au nanoparticles were identified using the concentric backscatter (CBS) detector at an accelerating voltage of 20 keV and beam current of 0.40 nA. To prevent ion-beam surface damage to the region of interest (ROI) during milling, reduce curtaining effects and strengthen the lamella, an in-situ protective layer of Pt 23-µm long, 3-µm wide and 2-µm thick was deposited using the GIS. Conventional cross-section patterns were used to excavate material from either side of the Pt layer to a depth of ~19 µm. Milling was performed at 30 keV with a stepwise lowering of the ion beam current from 65 nA to 0.79 nA. After thinning a lamella to a thickness of ~1 µm with an ion beam current of 0.79 nA, the lamella was lifted out and attached to a Cu TEM half grid using the Easylift nanomanipulator. Owing to the large size of the lamella (~23 x 19 µm) and hardness of the sample, the final steps were to thin both sides of the lamella to electron transparency with higher-than-normal beam energy: to ~500 nm using 30 keV and 0.79 nA, to 200 nm using 30 keV and 0.23 nA, and to ~100 nm using 30 keV and 80 pA.

High resolution imaging of the TEM lamellae was performed with a FEI Tecnai G2 F20 STEM (Thermo Scientific, Hillsboro, OR USA) equipped with an EDAX Octane T Ultra W /Apollo XLT2 SDD and TEAM EDS (energy-dispersive X-ray spectroscopy) Analysis System (EDAX, Inc. Mahwah, NJ USA) and a Fischione Instruments Model 3000 Annular Dark Field (ADF) Detector (E.A. Fischione Instruments, Inc., Export, PA USA). The machine was equipped with a Schottky field electron emitter and a TWIN objective lens. The TEM point resolution was 0.27 nm and the line resolution were 0.14 nm. The scanning transmission electron microscope (STEM) high angle annular dark field (HAADF) resolution was 0.3 nm. For STEM, the collection angle was 57 mrad, and the objective aperture was 100 micron A Gatan Ultrascan 4000 4k x 4k CCD Camera System Model 895 was used for TEM imaging, recording and processing including fast Fourier transformation (FFT) and inverse fast Fourier transformation (IFFT). Images were recorded in bright field mode at an accelerating voltage of 200 kV using the Gatan Model 895 Ultrascan 4000 CCD Camera System (Gatan, Inc., Warrendale, PA USA). STEM was performed by using a HAADF detector operating through the Tecnai imaging and analysis (TIA) interface.

EDAX analysis was supported with an Apollo XLT2 windowless silicon drift detector (SDD) operating through the TIA interface. Point analysis spectra (focussed probe) were recorded with this system.

Data Supplement #2 – Ancillary TEM Imagery

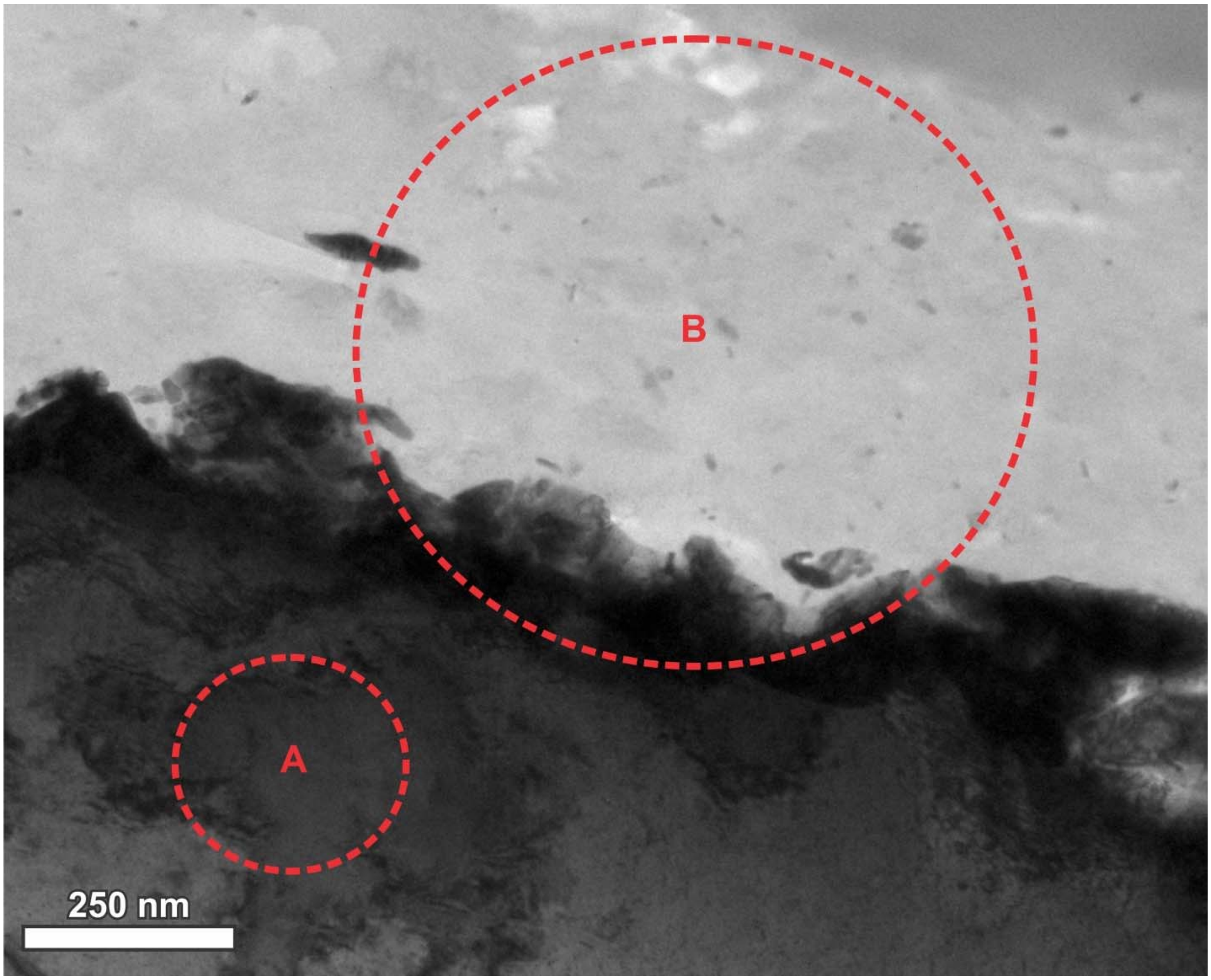

Figure S1: A bright field TEM image of the contact between a large electrum grain (lower half of image) and the calcite matrix of the host calcite-quartz vein (upper half of image) showing the source locations (A and B) of SAED images presented in Figures S2 and S3.

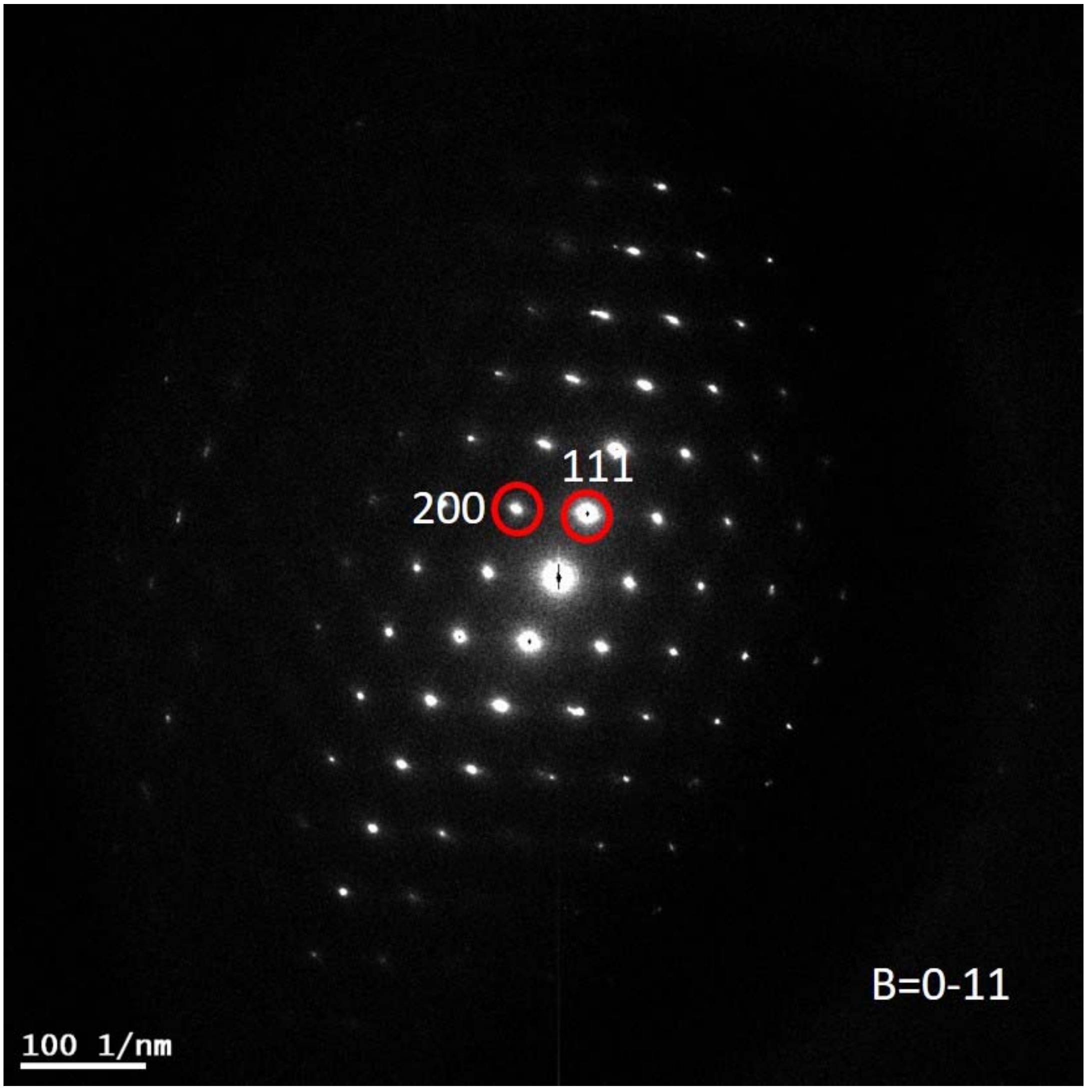

Figure S2: A selected area electron diffraction (SAED) image of area A in Figure S1. The diffraction maxima shown here are typical of those for monocrystalline gold. The reflection used to generate the image is (111).

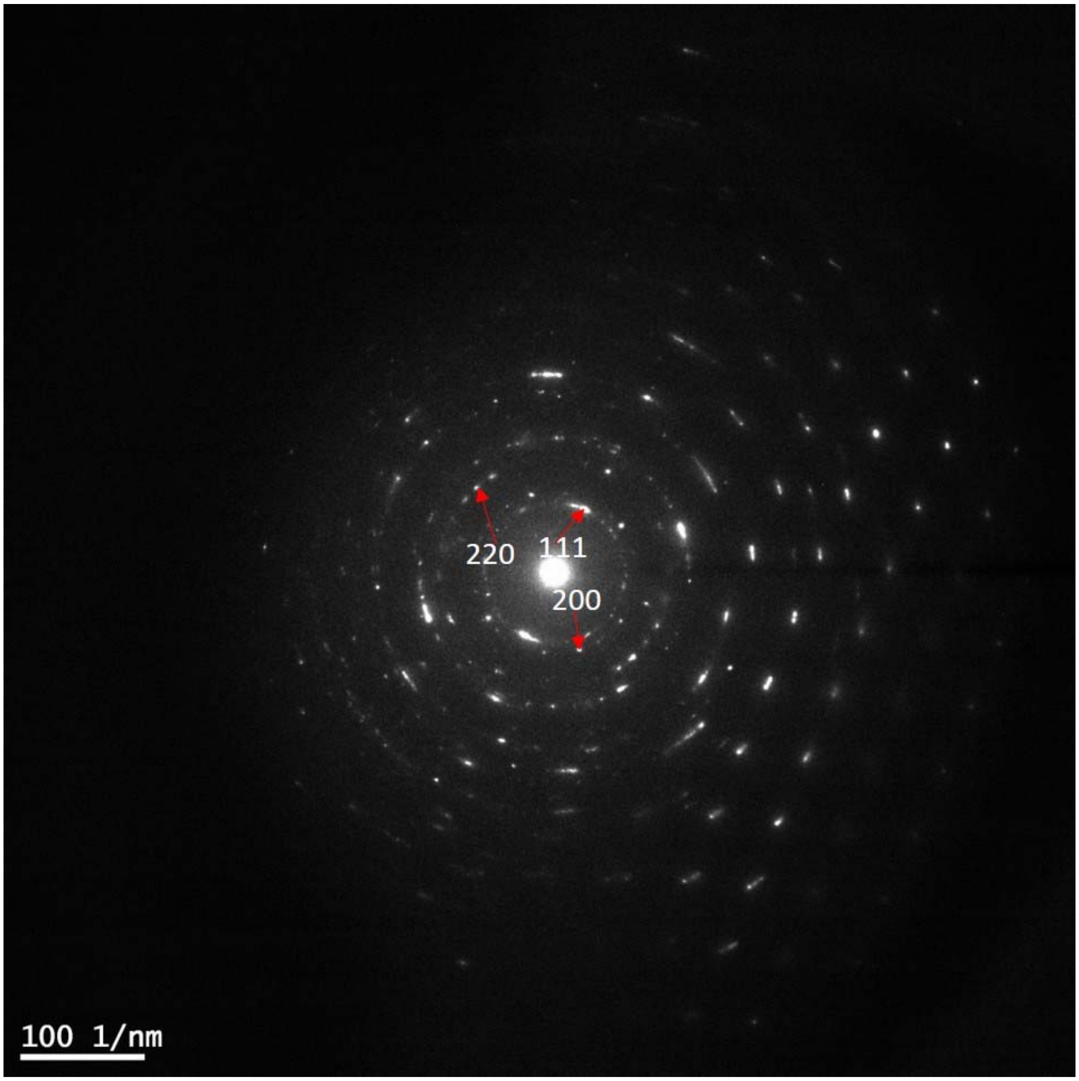

Figure S3: A selected area electron diffraction (SAED) image of area B in Figure S1. The diffraction maxima are typical of those for polycrystalline gold and indicate that multiple gold (electrum) crystals are present in the imaged area.

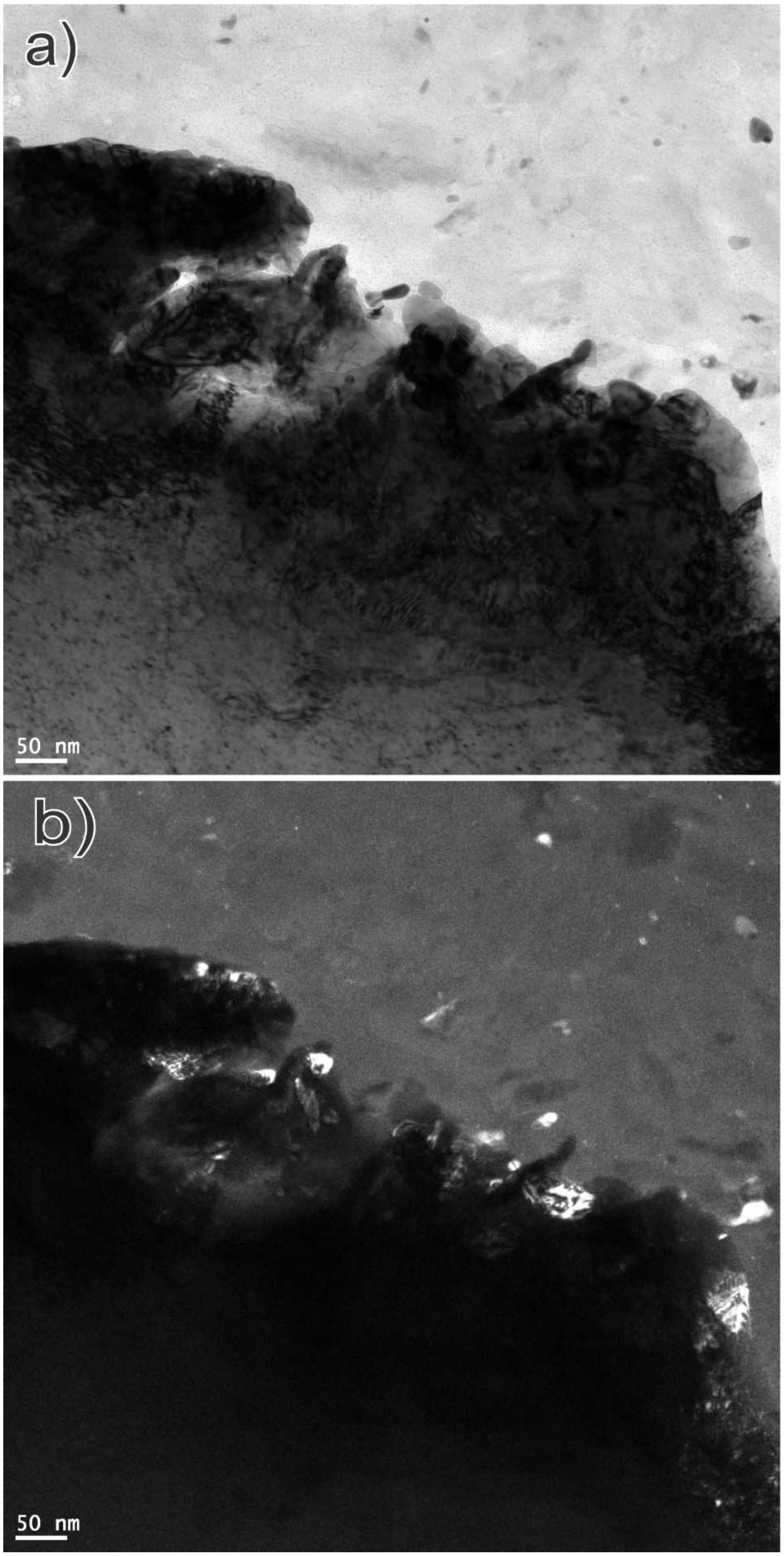

Figure S4: (a) bright field and (b) corresponding high-angle angular dark field (HAADF) TEM images of the contact between a large electrum grain (lower left in both images) and the matrix of a calcite vein, which contains nanoparticles of electrum (higher contrast spots in the matrix in the HAADF image).

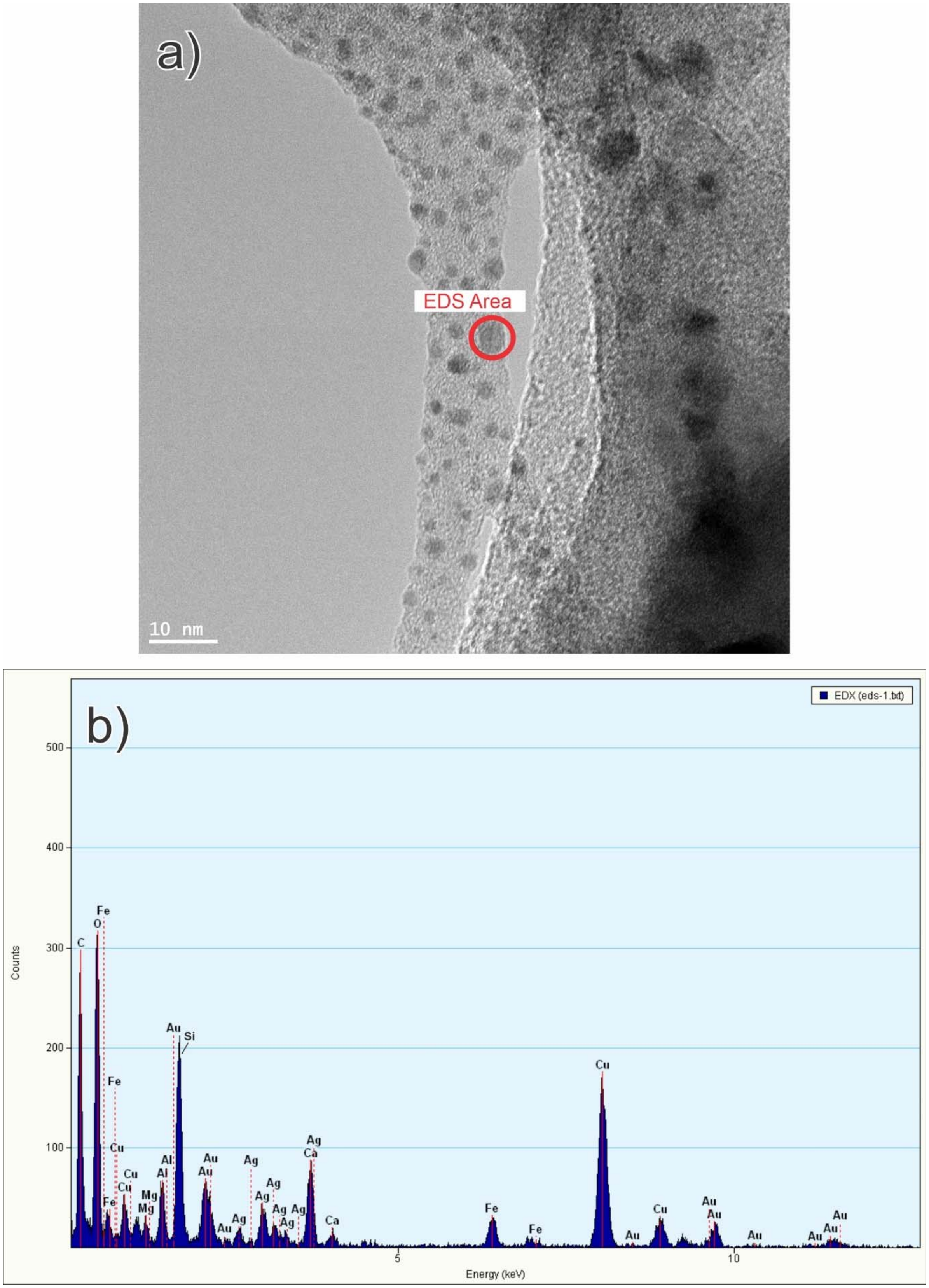

Figure S5: (a) bright field TEM image showing gold (electrum) nanoparticles in a calcite-chlorite matrix. (b) Results of an EDS analysis of the circled nanoparticle in the bright field image, confirming that it is electrum. The Cu peaks are artifacts from the instrument. The Ca, C, and O peaks confirm that the matrix material surrounding the nanoparticle is calcite with chlorite (Mg, Fe, Al, and Si peaks).
